# Disruption of NOTCH signaling by a small molecule inhibitor of the transcription factor RBPJ

**DOI:** 10.1038/s41598-019-46948-5

**Published:** 2019-07-25

**Authors:** Cecilia Hurtado, Alena Safarova, Michael Smith, Raeeun Chung, Arne A. N. Bruyneel, Jorge Gomez-Galeno, Franz Oswald, Christopher J. Larson, John R. Cashman, Pilar Ruiz-Lozano, Philip Janiak, Teri Suzuki, Mark Mercola

**Affiliations:** 1 Stanford Cardiovascular Institute and the Department of Medicine, Stanford University, Stanford, CA 94305 USA; 2 Sanford-Burnham-Prebys Medical Discovery Institute, La Jolla, CA 92037 USA; 3 Icagen, Oro Valley, AZ 85755 USA; 4 Human BioMolecular Research Institute, San Diego, CA 92121 USA; 5 University Medical Center Ulm, 89081 Ulm, Germany; 6 Regencor, Los Altos, CA 94022 USA; 7 Sanofi, 91380 Chilly-Mazarin, France; 8 Present Address: Belltree Consulting, L.L.C., Tucson, AZ 85745 USA

**Keywords:** Screening, Drug discovery

## Abstract

NOTCH plays a pivotal role during normal development and in congenital disorders and cancer. γ-secretase inhibitors are commonly used to probe NOTCH function, but also block processing of numerous other proteins. We discovered a new class of small molecule inhibitor that disrupts the interaction between NOTCH and RBPJ, which is the main transcriptional effector of NOTCH signaling. RBPJ Inhibitor-1 (**RIN1**) also blocked the functional interaction of RBPJ with SHARP, a scaffold protein that forms a transcriptional repressor complex with RBPJ in the absence of NOTCH signaling. **RIN1** induced changes in gene expression that resembled siRNA silencing of RBPJ rather than inhibition at the level of NOTCH itself. Consistent with disruption of NOTCH signaling, **RIN1** inhibited the proliferation of hematologic cancer cell lines and promoted skeletal muscle differentiation from C2C12 myoblasts. Thus, **RIN1** inhibits RBPJ in its repressing and activating contexts, and can be exploited for chemical biology and therapeutic applications.

## Introduction

NOTCH proteins are trans-membrane receptors that transduce signals from cell-bound JAGGED (JAG) and Delta-like (DLL) families of ligands to mediate cell-cell interactions in processes as diverse as fetal development, heart disease and cancer^1,2^. Upon interaction with ligand, NOTCH is cleaved by γ-secretase to release an intracellular domain (NICD) that binds the transcriptional effector RBPJ [recombination signal-binding protein for immunoglobulin κ J region, also known as CSL and CBF1]^3,4^. There is only one small molecule reported to selectively inhibit NOTCH signaling and none known to target RBPJ^5^. γ-secretase inhibitors (GSIs) have been used widely to block the proteolytic activation of NOTCH, but are inherently unselective since they also block the processing of >90 other substrates, including the amyloid precursor protein (APP), ErbB4, and E-cadherin^6–8^. Although newer generation GSIs exhibit a biased inhibition of APP over NOTCH^9,10^, there are no NOTCH-selective GSIs. Clinical use of GSIs cause numerous side effects, notably intestinal crypt cell proliferation^11^, and skin rashes and tumors^12^. These undesirable effects have led to the early termination of a phase III clinical trial of Semagacestat for treatment of Alzheimer’s Disease^13^.

The NOTCH ICD:RBPJ complex contains co-activators MAML (mastermind-like protein) and histone acetyltransferases (HATs) to activate downstream genes including members of the structurally related HES (Hairy/Enhancer of Split) and HEY/HRT (Hairy/Enhancer of Split-related) family^14^. The transcriptional effects of NICD are mediated primarily through the interaction with RBPJ, which recruits NICD to recognition sites in promoter regions as well as to more distally located superenhancers^15^. Thus, antagonism of RBPJ would be a desirable point to modulate NOTCH signaling, making it a useful probe and potential clinical candidate since it could provide additional selectivity over targeting γ-secretases or NOTCH itself. To develop a novel chemical inhibitor of NOTCH, we evaluated the feasibility of selectively targeting the RBPJ protein to perturb its interaction with the NOTCH ICD.

Although protein-protein interactions can be challenging to inhibit with small molecules, the binding interface between RBPJ and NOTCH is small^16,17^ hence we reasoned that it might be possible to disrupt it with a small molecule. To increase the likelihood of selectively targeting RBPJ (as opposed to NOTCH directly, or mediators of its expression, trafficking or cleavage), we developed a primary high throughput screen (HTS) aimed at disrupting the interaction between RBPJ and an unrelated scaffold protein, SHARP, that binds to the same region of RBPJ to mediate its repressive effects^18^. Hits from this screen were triaged by secondary screens that identified inhibitors of RBPJ in the context of NOTCH ICD. One molecule, termed **R**BPJ **IN**hibitor-1 (**RIN1**), potently disrupted the functional interaction of RBPJ with NOTCH and functioned as a probe of RBPJ function in cancer and skeletal myogenesis models. **RIN1** is the first inhibitor of RBPJ and can be exploited for research and possible therapeutic applications.

## Results

### Identification of an RBPJ inhibitor

**Figure 1:**
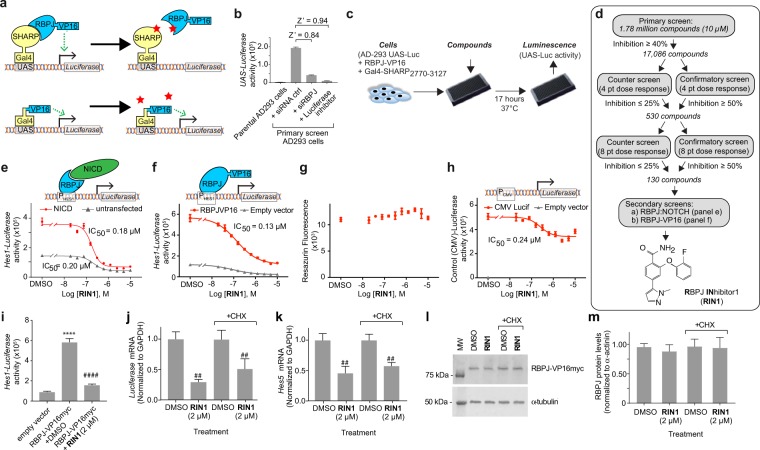
Identification of small molecule RBPJ inhibitor, RIN1. (**a**) Schematic of the primary and counter screens. The screen was a cell-based two hybrid assay in which an active compound (stars) would disrupt the SHARP:RBPJ interaction and decrease activity of the Luciferase reporter. A minimal RBPJ-interacting domain of SHARP and a DNA-binding mutant of RBPJ were used (see Methods). (**b**) Assay validation using RBPJ siRNA transfection and a small molecule Luciferase inhibitor, Data is mean ± standard deviation, n = 5 wells. Z’ is a metric of dynamic range^19^. (**c**) Workflow schematic. (**d**) Screen flowchart and structure of **RIN1**. (**e**,**f**) Inhibition of NOTCH2 ICD (**e**) and RBPJ-VP16myc fusion protein (**f**) activity on the *Hes1-*Luciferase reporter in transient transfections, n = 4 wells. (**g**) Effect on cell viability, n = 4 wells. (**h**) Effect on CMV promoter activity, n = 4 wells. Data in b-h are presented as mean ± standard deviation; experiments were repeated > 3 times. (**i**–**k**) Effect of Cycloheximide on **RIN1** inhibition of RBPJ-VP16. AD-293 cells were transfected with RBPJ-VP16myc and 48 hours later were treated ± **RIN1** (2 µM), ± Cycloheximide (CHX, 10 µg/ml) for an additional 17 hours and then assayed for *Hes1*-Luciferase activity (**i**) (n = 10 wells), *Luciferase* mRNA (**j**) and endogenous *HES5* mRNA (**k**), (n = 3 samples). Western blot of RBPJ-VP16myc fusion protein under identical conditions (**l**) and its quantification (**m**), n = 3 wells. Data are presented as mean ± standard deviation. Incrementing number of symbols (* and #) denote P < 0.05, P < 0.01, P < 0.001 and P < 0.0001 respectively, using two-tailed unpaired Student’s T-test relative to empty vector (*) or to RBPJ-VP16myc + DMSO vehicle control (#) conditions. Experiments were repeated twice.

To identify selective inhibitors of RBPJ, we developed a primary screen to detect inhibitors of a functional interaction between RBPJ and the scaffold protein SHARP that was followed by secondary assays to establish efficacy against NOTCH. The primary screen was adapted from a cell-based 2-hybrid assay that probed the interaction of RBPJ with a minimal RBPJ-binding fragment of SHARP (amino acid residues 2770–3127)^18^ (Fig. 1a, upper schematic). To minimize unwanted genomic effects of RBPJ, we used a mutated form of RBPJ that cannot bind to DNA (see Supplemental Methods). A functional interaction between the two proteins activated the UAS-Luciferase reporter gene in stably transfected AD-293 cells. We used siRNA against RBPJ to determine that the assay had an acceptable dynamic range (Z’ = 0.84) (Fig. 1b). Z’ scores between 0.5 and 1.0 are considered excellent^19^.

The primary assay was screened in 1536-well format against 1,780,000 compounds from the Sanofi Tucson combinatorial collection and the Sanofi Screening Collection (SASC1) at a single concentration (10 µM) (Fig. 1c and Supplementary Table 1). Using a single hit compound from a pilot screening at the Prebys Center for Drug Discovery (La Jolla, California, USA), we determined that the mean Z’ for the entire screen was 0.64 ± 0.05. The hit selection cutoff was set at 4 standard deviations of the mean of all control wells across the entire screen, which corresponded to a threshold of ≥40% inhibition of UAS-Luc activity and yielded 18,047 compounds. Some of these were either duplicates or unavailable for retesting, therefore 17,086 primary positives were retested through a dose range to confirm activity (Fig. 1d). To rule out inhibition of the Luciferase reporter or other non-specific effects on the cell assay system, hits were also tested in a counter screen that was identical to the primary assay except that a fusion protein consisting of the Gal4 DNA binding domain covalently linked to the VP16 activation domain was used instead of the separate Gal4-SHARP and RBPJ-VP16 fusion proteins in the primary assay (Fig. 1a bottom schematic). Of the primary assay hits, 530 compounds confirmed activity (≥50% inhibition at 10 µM or lower concentration) in the confirmatory assay *and* were considered inactive (≤25% inhibition) in the counter screen. Of these compounds, 130 showed dose responsive inhibition comprising 14 distinct chemical families plus 17 singletons.

To distinguish compounds that inhibit RBPJ (as opposed to SHARP), we designed a secondary assay to test whether the molecules would block the function of RBPJ in the context of activated NOTCH signaling. In this assay (Fig. 1e), NOTCH2 ICD was transiently expressed in AD-293 cells harboring a *Hes1-*Luciferase reporter construct. One compound, **RIN1**, inhibited *Hes1*-Luciferase activity with an IC_50_ of 0.18 µM and E_max_ of 82% (Fig. 1e). **RIN1** also inhibited NOTCH3 ICD with similar potency and efficacy (0.19 µM and E_max_ = 88%). We tested whether **RIN1** would inhibit a RBPJ-VP16 fusion protein that, because of the Herpes Simplex Virus VP16 transactivation domain, induces transcription independently of NICD and co-activators. Again, **RIN1** inhibited RBPJ-VP16-dependent *Hes1-*Luciferase with the same potency and efficacy (IC_50_ = 0.20 µM and E_max_ = 81% inhibition; Fig. 1f). In the same experiment, **RIN1** had only a minimal effect on cell viability (Resazurin) (12% inhibition at 10 µM, Fig. 1g) or on the CMV promoter (22% reduction at 10 µM, Fig. 1h) that was used to direct transgene expression in the primary and secondary assays. Thus, these data suggest that **RIN1** is a potent inhibitor of RBPJ in the contexts of NICD and SHARP signaling.

Next, we examined if **RIN1** would inhibit pre-existing RBPJ or if it must be present during synthesis for inhibition to occur. If **RIN1** were required during RBPJ synthesis, its inhibitory activity should be abrogated by treatment with cycloheximide, which blocks translation of nascent proteins. **RIN1** (2 µM) decreased RBPJ-VP16-dependent *Hes1-*Luciferase activity and caused a corresponding decrease in *Luciferase* reporter mRNA and endogenous RBPJ target gene expression (Fig. 1i–k) mRNAs. Cycloheximide did not abrogate the ability of **RIN1** to inhibit either *Luciferase* or endogenous gene expression (Fig. 1j,k). Furthermore, **RIN1** did not alter the abundance of RBPJ-VP16 protein (Fig. 1l,m) under these conditions. Similarly, when new synthesis was blocked by siRNA silencing in cells, RIN1 did not alter the decay kinetics of endogenous RBPJ (Fig. S1). These data suggesting that RIN1 does not alter the turnover of RBPJ. Instead, the ability to inhibit in the presence of cycloheximide suggests that **RIN1** disrupts the function of pre-synthesized RBPJ.

### RIN1 inhibits NOTCH-dependent tumor cell proliferation

**Figure 2:**
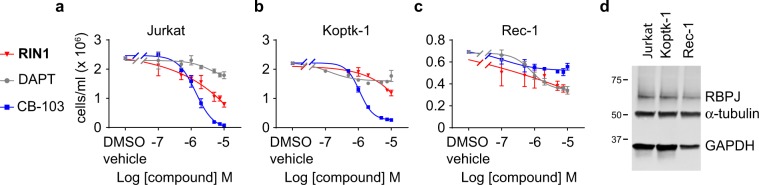
Comparative effects of RIN1, DAPT and CB-103 on hematologic tumor cell proliferation. (**a**–**c**) Acute T cell leukemia cell lines Jurkat (**a**) and KOPT-K1 (**b**) and non-Hodgkin’s mantle cell lymphoma Rec-1 line (**c**) were treated with small molecule NOTCH inhibitors during their logarithmic growth phase as indicated for 96 hours. n = 7, assay repeated twice. (**d**) Western blot showing levels of RBPJ in the tumor cell lines.

NOTCH plays a role in the carcinogenesis and tumor progression, including leukemia, breast and lung cancers^2^. As a bioassay of NOTCH inhibition, we assayed the effect of small molecule NOTCH inhibitors on the proliferation of two cell lines immortalized from T-cell acute lymphoblastic leukemia (T-ALL) patients (Jurkat and KOPT-K1) and in the mantle cell lymphoma (MCL) line REC-1, all of which have activating mutations in the NOTCH1 heterodimerization and/or PEST domain common in hematologic malignancies^20,21^. **RIN1**, the γ-secretase inhibitor DAPT, and CB-103, a recently described small molecule NOTCH inhibitor with no reported mechanism^22^, all decreased cell proliferation in the three cancer cell lines but with markedly different potencies and efficacies (Fig. 2a–c). The tumor lines had comparable levels of RBPJ protein (Fig. 2d); therefore, the varying effects of **RIN1** on tumor cell proliferation might reflect differential reliance on RBPJ-dependent versus RBPJ-independent NOTCH signaling^23^.

### RIN1 promotes muscle differentiation

**Figure 3:**
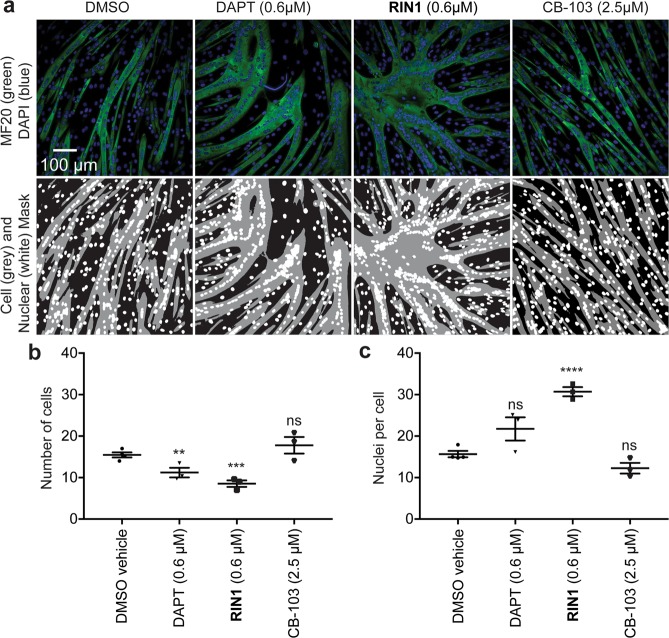
Effect of RIN1 on C2C12 myoblast differentiation. Structured illumination photomicrographs of C2C12 cells at 4 days under permissive differentiation conditions and drug treatment as indicated. Upper panels: Cells were stained for myosin heavy chain with the MF20 antibody (green) and labeled with DAPI to identify nuclei (blue) (upper panels). Lower panels: Cell body and nuclei image masks for quantification. (**b**,**c**) Image analysis, n = 3 wells, quantified the number of cell mask (green) objects (**b**) and the ratio of nuclei per cell mask object (**c**). Assay repeated 3 times.

As a second bioassay of NOTCH inhibition, we tested the function of the small molecule inhibitors on the differentiation of muscle progenitor cells into mature myofibers, which is blocked by endogenous NOTCH activation^24^. C2C12 myoblasts were induced to differentiate by passaging from low density, high serum culture conditions into high density, low serum conditions causing fusion of the myoblasts into multinucleated myofibers that expressed structural muscle proteins such as α-actinin and myosin heavy chain (MHC). Fusion was quantified by image analysis measuring the number of MHC^+^ cells and the number of nuclei per cell (Fig. 3). Relative to treatment with DMSO vehicle alone, **RIN1** (0.6 µM, corresponding to 3 × IC_50_) decreased the number of MHC^+^ cells and increased the number of nuclei per cell, indicating that it induced the formation of multinucleated myofibers. DAPT (0.6 µM, ~3 × IC_50_) was less potent in this assay, and CB-103 (0.6 or 2.5 µM) did not affect the formation of myofibers. Thus, **RIN1** was active in both the cancer cell anti-proliferation and myoblast differentiation assays.

### RIN1 treatment resembles RBPJ silencing

**Figure 4:**
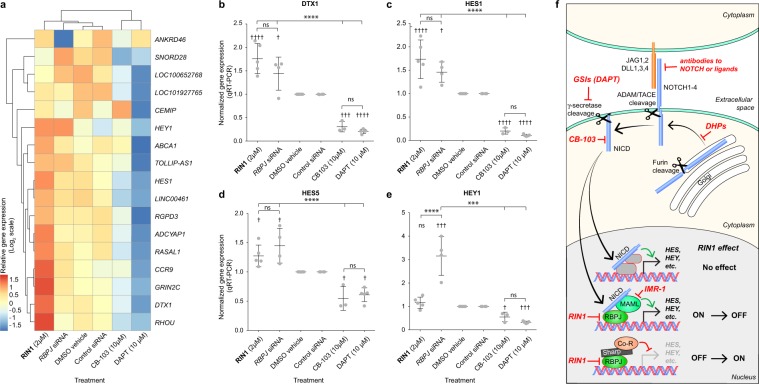
Gene expression changes induced by RIN1. Jurkat cells were treated with either 8 hours with small molecules or 48 hours with transfected *RBPJ* siRNA and DMSO-vehicle or control (inert sequence) siRNA, respectively. The heatmap represents changes in the levels of transcript (fold change > 2, P < 0.05) that were induced by either the small molecules or RBPJ siRNA relative to their respective controls (DMSO vehicle or control siRNA) and varied < 40% between techniques (DMSO-vehicle vs. control siRNA. (**b**–**e**) qRT-PCR analysis of RBPJ target gene expression. Data are presented as mean ± standard deviation. Incrementing symbols (* and †) denote P < 0.05, P < 0.01, P < 0.001 and P < 0.0001 respectively, using two-way ANOVA with Dunnett’s post-test. *between groups indicated; ^†^relative to respective control treatments (DMSO or control siRNA). ns, not significant. (**f**) Schematic showing NOTCH pathway inhibitors (red text) in relationship to signaling and summary of RIN1 effects on downstream gene expression.

We expected that inhibition of NOTCH signaling at the level of RBPJ would have a different effect on gene expression than would disruption at the level of γ-secretase since RBPJ can both activate and repress NOTCH target genes^25^ and γ-secretase cleaves numerous proteins in addition to NOTCH^6–8^. Therefore, we compared the transcriptomic effects of treating Jurkat cells with **RIN1** (2 µM) to treatment with DAPT (2 µM), CB-103 (10 µM) (all compounds were at ~10x IC_50_) and siRNA against *RBPJ*, which reduced Jurkat cell proliferation consistent with the effect of **RIN1** (Fig. S2). To compare gene expression profiles, we analyzed transcripts that did not vary (fold change <1.4, p < 0.05) between negative control groups (DMSO vehicle for the small molecules versus control siRNA for siRNA against RBPJ) to remove from analysis any genes that were influenced by the treatment differences *per se* (siRNA versus small molecule). Transcripts that were differentially expressed (fold change ≥ 2, p < 0.05) are shown in the heatmap (Fig. 4a**)** and representative examples were confirmed by qRT-PCR (Fig. 4b–e). DAPT repressed known target genes as expected, including *HES1*, *HEY1* and *DTX*. CB-103 resembled DAPT suggesting that it functions at the level of inhibiting NOTCH. In contrast, **RIN1** resembled siRNA silencing of RBPJ, albeit often with a greater response, suggesting that it acts at the level of RBPJ. The differential response of NOTCH target genes to **RIN1** and RBPJ siRNA compared to DAPT and CB-103 suggests that NOTCH target gene expression in Jurkat cells is simultaneously sustained by RBPJ-independent signaling and repressed by RBPJ. Figure 4f positions **RIN1** in the context of NOTCH signaling and in relationship to other NOTCH pathway modulators.

## Discussion

Here we describe **RIN1** as the first small molecule inhibitor of RBPJ signaling. RBPJ can either activate genes by forming a complex with the NOTCH ICD when NOTCH is active, or silence an overlapping but non-identical set of genes by recruiting co-repressors in the absence of NOTCH signaling^25^. In the context of NOTCH signaling, RBPJ mediates many processes, including progenitor renewal and cell fate selection during embryogenesis and tissue homeostasis, as well as pathological processes such as tumorigenesis and pulmonary hypertension^1,2,26,27^. In addition to mediating NOTCH signaling, RBPJ can act independently, for instance to attenuate hypoxia signaling through direct physical interaction with HIF1α and 2α proteins^28^. **RIN1**, therefore, is a unique chemical tool to probe this biologically important protein.

**RIN1** treatment induced a profile of transcript changes that was distinct from the changes induced by DAPT, including opposing effects on bonafide NOTCH target genes *HES1*, *HES5*, *HEY1* and *DTX* (Fig. 4). Activation of some NOTCH target genes by RBPJ inhibition was not unexpected since RBPJ functions as both a repressor (in the absence of NOTCH signaling) and as an activator in response to NOTCH (Fig. 4f). **RIN1** was identified based on the functional inhibition of RBPJ complexed with SHARP (primary screen) and secondarily based on inhibition of NOTCH (secondary screen), with which it forms repressing and activating complexes, respectively. Furthermore, siRNA-mediated knockdown of RBPJ induced a similar profile of transcript changes as did **RIN1**. Together, we conclude that **RIN1** inhibits RBPJ in both its activating (NOTCH) and inhibiting (SHARP) complexes. In contrast, CB-103 induced a profile of gene expression changes that differed from that of **RIN1** but resembled the changes induced by DAPT, suggesting that CB-103, whose target is unknown, might act at or near the level of the NOTCH receptor itself. Consistent with targeting RBPJ, **RIN1** inhibited the activity of RBPJ-VP16 fusion protein that is a constitutively active transcriptional activator (Fig. 1f). Moreover, **RIN1** was not selective for NOTCH isoform, also consistent with it targeting RBPJ. Thus, the transcriptomic and functional data suggest that **RIN1** targets RBPJ itself or a closely interacting protein. Studies to evaluate the possible physical interaction with RBPJ are underway.

As examples of its utility as a chemical probe, **RIN1** suppressed the proliferation of three hematologic tumor cell lines (Jurkat and KOPT-K1 T-ALL, and REC-1 MCL). Interestingly, the potencies and efficacies of **RIN1** relative to DAPT and CB-103 varied across lines (Fig. 2). **RIN1** and siRNA against RBPJ effectively blocked proliferation of all cells; however, DAPT modestly blocked proliferation of Jurkat cells (Fig. 2), suggesting that RBPJ plays a relatively more important role in controlling Jurkat cell proliferation than does NOTCH cleavage and release of ICD. Also, CB-103 was more effective than either DAPT or **RIN1** in Jurkat and KOPT-K1, but not Rec1 lines, suggesting differential roles of the NOTCH pathway components across cancer lines with activating NOTCH mutations. **RIN1** also promoted the differentiation of the C2C12 skeletal myoblasts into multinucleated myofibers (Fig. 3). CB-103 was ineffective in this context suggesting that its target (which is unknown) is not involved, again illustrating the variable effects of inhibiting different NOTCH pathway components.

In summary, **RIN1** inhibits the functional association of RBPJ with SHARP and NOTCH ICD, thereby blocking the transcriptional repression and activation, respectively, of downstream genes. **RIN1** is structurally and functionally distinct from existing small molecule NOTCH inhibitors. The only previously known selective inhibitor, IMR-1, acts at the level of NOTCH itself^5^ (Fig. 4f). Other small molecule inhibitors (Fig. 4f) are γ-secretase inhibitors (GSIs), which act unselectively to block NOTCH processing, dihydropyridine (DHP) inhibitors of NOTCH trafficking^29,30^ and CB-103^22^, which appears to function at the level of NOTCH (Fig. 4) and has recently entered clinical development for treatment of NOTCH-dependent cancers^31^. As the first small molecule RBPJ inhibitor, **RIN1** could be exploited for chemical genetics and therapeutic applications.

## Methods

### HTS for small molecule RBPJ inhibitors

#### Screen and counter screen cell lines

The cell line AD-293 (Agilent # 240085) was used to generate a stable cell line carrying the UAS-Luciferase reporter plasmid pGL4.35 (Promega) by hygromycin selection (50 µg/ml) termed AD-293-UAS-Luc. Subsequently, this line was transfected (followed by clone selection) with the plasmids indicated below to generate the primary screen and counter screen lines. AD-293-UAS-Luc and its derivatives were grown in DMEM 4.5 g/ml glucose, sodium pyruvate, glutamine, pen/strep, 10% fetal bovine serum and selection antibiotic. Assay media was DMEM (Cellgro #17-205-CV) 4.5 g/ml glucose, glutamine, pen/strep, 5% fetal bovine serum, without phenol red.

To generate the primary screen cell line, AD-293-UAS-Luc cells were transfected with plasmid pBI-CMV (Clonetech) that contained two expression cassettes. Multiple cloning site 1 (ClaI-EcoRV) contained the Gal4 DNA binding domain (Gal4DBD) in frame with the nucleotide sequence corresponding to SHARP amino acids 2770–3127, which is a minimal fragment that retains RBPJ binding^18^. Multiple cloning site 2 (BglII-XbaI) contains the sequence of full-length human RBPJ containing 4 mutations that block binding to recognition sites in DNA^32^ (to avoid any effects of RBPJ directly interacting with DNA) fused to the transactivating domain of Herpes simplex virus VP16 and a MYC epitope tag.

To generate the counter screen cell line, AD-293-UAS-Luc cells were transfected with a plasmid that contained the CMV promoter driving the GAL4 DBD fused to the VP16 transactivation domain. This plasmid was made by replacing the DsRed2 coding region (AgeI-NotI fragment) in pDsRED2-C1 (Clontech) with a bicistronic DNA fragment encoding Gal4 DBD-VP16 separated from a UAS-eGFP cassette by a stop codon and poly adenylation sequence. This sequence was derived from pBSEGVUG obtained from Scott Fraser, Caltech.

#### High throughput (primary) screening

HTS was performed in white tissue culture treated 1536 well plates (Corning Cat. #3727), 500 cells per well in 5 µl volume. Compounds were dispensed by either Labcyte acoustical dispenser (100,000 compound pilot screen) or pintool (1.78 MM compound screen) and incubated 17 hours at 37 °C and 5% CO_2_. Luciferase substrate was Britelite Plus (Perkin Elmer). The assay had excellent properties (Z’ = 0.74 ± 0.06; S/B = 153 ± 19; hit rate of 0.16%, Supplementary Table S1) determined in a single point determination (10 µM) pilot screen of ~100,000 diverse small molecules at the Prebys Center for Drug Discovery (PCDD, La Jolla, CA) that yielded 15 structurally similar compounds after counter screening. After transfer to Sanofi-Tucson, the primary screen was performed identically against 1,780,000 compounds (10 µM) using the PCDD hit as a positive control and DMSO vehicle as negative control. The mean Z’ of the entire screen was 0.64 ± 0.05 with a 1% primary hit rate. Hits were evaluated through two confirmatory screens (Supplementary Table S1**)**, first 4-point (10, 2.5, 0.625, and 0.156 µM) and then 8-point (20, 10, 5, 2.5, 1.25, 0.625, 0.313, 0.156 µM) in the same assay as used for primary screening, and concurrently in counter screens that measured inhibition of luciferase constitutively driven by Gal4-VP16 (same concentration ranges).

### Secondary screening

#### *Hes1*-luciferase reporter line

The UAS sequence in pGL4.35 was replaced by the murine *Hes1* promoter (−194 to +160 relative to TSS of mouse Hes1 gene)^33^ to generate the Hes1-Luciferase reporter construct that was then stably transfected into AD-293 cells to yield AD-293 *Hes1*-Luciferase line. This cell line was transiently transfected in 384 well plates (reverse transfection) with plasmids to direct expression of NOTCH2 ICD (pAdloxN2ICD^28^), NOTCH3 ICD (pCDNA3.1 + Hygro N3ICD-HA^28^) and RBPJ-VP16myc fusion protein (pCDNA3.1 RBPJ-VP16-myc^28^). Luciferase signal was read out as for primary screening using the steadylite plus substrate (Perkin Elmer).

#### Secondary screen controls

The parental AD-293 cell line was transfected with plasmid pCNDA3.1-Firefly Luciferase [made by transferring the Firefly Luciferase gene from pGL3 (Sigma-Aldrich)]. For cell viability, resazurin (Sigma R7017) was added to the cells at 5 µg/ml final concentration and incubated 2 hours at 37 °C before fluorescence signal detection (Perkin Elmer Envision plate reader).

#### AD-293 transfections and western blot analyses

AD-293 were transfected with RBPJ siRNA (Dharmacon J-007772-06) or non-targeting control (D-001810-01) using Lipofectamine RNAiMax with the following protocol per 4 cm^2^ well (12 well format): 100 µl Optimem containing 1.2 ul siRNA at 25 µM was combined with 100 µl Optimem containing 3 µl Lipofectamine and briefly vortexed. After 5 minutes incubation the mixture was added to 550 ul complete culture media containing 330,000 cells and 250 µl media 8 µM compound (or DMSO). Final siRNA concentration was 30 nM and final compound concentration was 2 µM. After 12, 24 or 48 hours of culture, cells in wells were rinsed with cold PBS and lysed with 175 µl RIPA buffer containing protease inhibitor cocktail. Protein concentration was measured with BCA protein assay (Pierce). 6 µg of total protein was loaded per well in polyacrylamide gel (Miniprotean TGX 10% gel) and transferred to PVDF membrane. Antibodies used were: anti-RPBJ (Cell Signaling 5313, rabbit) and anti-α-Tubulin (T5168, mouse). Secondary antibodies were 780 nm anti-rabbit and 680 nm anti-mouse Ig. Imaging and band quantification were done using an Odyssey Licor System.

### Cellular assays

#### Tumor cell proliferation assays

Jurkat, Koptk-1 and Rec-1 cells were grown in RPMI 1640 supplemented with glutamine, pyruvate, 10% fetal bovine serum and pen/strep. The proliferation assay was performed in black wall, clear bottom 384 well plates. Each well received 20 µl of cell suspension and 20 µl of media with compound (at 2x final concentration). 48 hours later, an additional 40 µl of media with 1x compound was added to each well. At 96 hours of culture, Resazurin was added to each well and cell number was calculated from a standard curve of Resazurin fluorescence intensity as a function of cell number for each cell type.

#### C2C12 myoblast differentiation assay

C2C12 cells were grown at low density in DMEM 4.5 g/l glucose, pen/strep and 10% FBS. Cells were seeded in 96 well plate black wall clear bottom at 10,000 cells per well in DMEM 20%FBS. Media was removed on the following day, and wells were rinsed once with PBS and replaced with 200 µl differentiation media (DMEM 2% horse serum) plus treatment (considered day 0). Three wells were assigned to each treatment and DMSO concentration was 0.1% in all samples. Cells were incubated under 3% oxygen (5% CO_2_, 92% N2). Media (+treatment) was replaced at day 1 and 2 (media changes for hypoxia conditions were done under 5% oxygen). On day 4 (90 hours from initial treatment) cells were fixed with 4% paraformaldehyde for 10 minutes. MHC immunostaining was done using MF20 antibody concentrate (Developmental Studies Hybridoma Bank) overnight at 1:100 dilution in blocking buffer (3% BSA, 0.1% TritonX100, 0.2% Glycine PBS). Secondary antibody anti mouse Alexa 488 was used at 1:200 dilution. Nuclei were counter-stained using DAPI and cells were imaged using 20× (0.75 N.A.) objective using an IC200 automated microscopy system (Vala Sciences, San Diego, CA).

### Image analysis

DAPI and Alexa488 image Z-stacks image stacks consisting of 20 images with step size of 1 µm were obtained by structural illumination microscopy using an IC200 automated microscopy system (Vala Sciences, San Diego, CA) at 20× (0.75 N.A.). Each stack was then projected (maximum intensity projection) and analyzed to calculate the following parameters in ImageJ/Fiji. Nuclei count: A nuclear mask and count was created from the DAPI images by size thresholding (400 pixels) using the Analyze Particle function in ImageJ. MHC^+^ object count: The MHC^+^ object mask (to identify cells) was created as for the nuclear mask but using the Alexa488 images and a minimum size threshold of 3000 pixels and a constant signal intensity threshold for all images. This value is reported in Fig. 3b as the “Number of cells”. MHC^+^ nuclei count: MHC^+^ object and nuclei masks were overlaid. Nuclear objects with >80% MHC signal were counted as MHC^+^ nuclei. Average nuclei count per MHC^+^ object: This value is calculated as the ratio MHC^+^ nuclei/MHC^+^ objects) and reported in Fig. 3c as “Nuclei/cell”.

### qRT-PCR

Total RNA was extracted using Quick-RNA miniprep kit (Zymo Research) following kit protocol including a DNAse treatment step. RNA was quantified using a Nanodrop and 500 ng of total RNA was used for reverse transcription, which was performed with the Quantitect RT klt (Qiagen), which includes a genomic DNA removal step. cDNA was diluted to avoid PCR inhibition by contaminants. 0.2 µl of cDNA was used per 10 µl of qRT-PCR reaction for all genes tested except for *HES5* that required 2 µl of cDNA per 20 µl qRT-PCR reaction. qRT-PCR reactions were performed using iTaq Universal SYBR Green (BioRad) in ABI 7900HT (Applied Biosystems) following manufacturers’ protocols.

### RNA sequence analysis

Jurkat cells were transfected with siRNAs by electroporation (Neon, Thermo Fisher Scientific) using the following conditions: 100 µl cells at 20 million cells/ml, 10 µl siRNA at 100 µM, 3 pulses of 10 msec duration at 1350 V. siRNAs used were: Dharmacon J-007772-06 (CUCCCAAGAUUGAUAAUUA; for RBPJ NM_203283), non-targeting control D-001810-01 and siGLO (D-001630-02) to assess transfection efficiency.

RNA sequencing was performed by Novogene (Illumina HiSeq) to obtain 20 million paired-end ~150 bp reads per sample in biological duplicate. FasQC (v0.11.5) and MultiQC (v1.3) were used to assess read quality. Adapter and quality trimming of reads were performed with Trimmomatic (v0.36). Reads were mapped to genome GRCh37 (hg19) using STAR (v2.5.3a) with UCSC gene annotations, and on average 19.1 million uniquely mapped reads were counted. Uniquely mapped reads were summarized at the gene level with featureCounts (v1.28.1) from the Rsubread module. Differential expression was performed with DESeq2 (v1.17.39). Genes having a fold-change of greater than 1.5 and a p-value of less than 0.05 were considered significant. Analysis filters are described in the text and legend to Fig. 4.

### Statistical analyses

Statistical analyses were performed on GraphPad Prism software using two way ANOVA with Dunnett’s post-test or by an unpaired Student’s T-test for calculation of P-values as indicated in the figure legends. The primary and counter screens were performed at a single dose and determination for each compound. The initial confirmatory screen was performed at 4 doses, single determination each, per compound. The second confirmatory screen was performed at 10 doses, single determination for each compound. The secondary screens and subsequent studies using **RIN1** were repeated with multiple determinations each (n indicated in the figure legends) and a minimum of three times with similar results. All experiments were repeated as indicated in the figure legends.

## Supplementary information

Supplementary Information

## Data Availability

The Jurkat cell RNAseq datasets are available on NCBI GEO (Accession number GSE134401). All other data generated or analyzed during this study are included in this published article (and its supplementary information files).
