# Supplementary material for: Disruption of NOTCH signaling by a small molecule inhibitor of the transcription factor RBPJ
Source: Sci Rep. 2019 Jul 25;9:10811. doi: 10.1038/s41598-019-46948-5 (PMC6658660; doi:10.1038/s41598-019-46948-5)
Supplement: Supplementary file 1 — Supplementary Information [file 41598_2019_46948_MOESM1_ESM.docx]

# Supplementary Information

**Disruption of NOTCH signaling by a small molecule inhibitor of the transcription factor RBPJ**

Cecilia Hurtado, Alena Safarova, Michael Smith, Raeeun Chung, Arne A.N. Bruyneel, Jorge Gomez-Galeno, Franz Oswald, Christopher J. Larson, John Cashman, Pilar Ruiz-Lozano, Philip Janiak**,** Teri Suzuki and Mark Mercola

**Contents Pages**

Table S1 2-3

Figures S1 and S2 4

Uncropped version of western blots 5-7

## Table S1. Overview of the screen.

| ***Category*** | ***Parameter*** | ***Description*** |
| --- | --- | --- |
| ***Assay*** | Type of assay | Cell-based two hybrid |
|  | Target | RBPJ |
|  | Primary measurement | Luciferase activity |
|  | Key reagents | AD-293 cells modified to include UAS-Luc reporter and RBPJ_DNA binding mutant_-VP16 GAL4-SHARP_2770-3127_ expression cassettes. |
|  | Assay protocol | 500 cells/well were plated onto pre-spotted compound in 1536-well plates. 17 hours later, cells were lysed and Luciferase activity determined using Bright Lite Plus (Perkin Elmer) |
|  | Additional comments | Baseline luciferase signal depends on the interaction between RBPJ and the SHARP_2770-3127_ fragment |
| ***Library*** | Library size | 1.78 million |
|  | Library composition | Combinatorial Collection (1.1m)  Historical Collection (680,000) |
|  | Source | Sanofi |
|  | Additional comments | none |
| ***Screen*** | Format | 1536 luciferase reporter assay |
|  | Concentration(s) tested | 10 µM |
|  | Plate controls | No compound (negative) control and positive control (10µM) from pilot screen at Prebys Center for Drug Discovery |
|  | Reagent/ compound dispensing system | Automated pintool |
|  | Detection instrument and software | EnVision multimodal plate reader  Version 1.13 |
|  | Assay validation/QC | Assay validated by siRNA silencing of RBPJ-VP16 (which mimics the effect of desired hits and verifies dependence on RBPJ) |
|  | Correction factors | None |
|  | Normalization | Positive controls |
|  | Additional comments | None |
| ***Post-HTS analysis*** | Hit criteria | 4 standard deviations from mean of control wells (40% inhibition) |
|  | Hit rate | Primary hit rate: 1%, Rate of confirmation for primary positives: 0.33% |
|  | Additional assays  Confirmatory 1:  Confirmatory 2:  Counter screen:  Hit criteria for Confirmatory & Counter screens:  Secondary 1:  Secondary 2: | 4-point (10, 2.5, 0.625, and 0.156 µM) in the same assay as used for primary screening  8-point (20, 10, 5, 2.5, 1.25, 0.625, 0.313, 0.156 µM) in the same assay as used for primary screening  Inhibition of luciferase constitutively driven by Gal4-VP16 (same dose range as for Confirmatory 1 and 2)  ≥ 50% inhibition 10µM or lower dose in the confirmatory screens 1 & 2 *and* ≤ 25% inhibition in the counter screen  10-point (10 µM plus 2-fold dilution series to 0.2 nM) of compounds screened for inhibition of NOTCH2 ICD-dependent *Hes1*-luciferase activity (Fig. 1e).  10-point (10 µM plus 3-fold dilution series to 0.2 nM) of compounds screened for inhibition of RBPJ-VP16myc-dependent *Hes1*-luciferase activity (Fig. 1f). |
|  | Confirmation of hit purity and structure | Purity and correct molecular weight were confirmed by LC-MS |
|  | Additional comments | Additional assays confirmed negligible effect on cell viability or CMV-luciferase activity (Figs. 1g,h) . |

Supplementary Fig. S1

**Fig. S1. RIN1 effect on RBPJ protein decay.**

1. Decay of endogenous RBPJ protein (normalized to α-tubulin) in HEK293T cells in response to treatment with RIN1 (2µM) relative to DMSO vehicle alone for up to 48 hours. Protein levels were determined from western blots (**b**).
2. Western blots. D, DMSO vehicle; R, **RIN1** (2µM).

## Supplementary Fig. S2

**Fig. S2. siRNA against RBPJ attenuates growth of Jurkat cells.**

Jurkat cells electroporated with siRNA against RBPJ or with control siRNA and seeded at 0.5 million cells/ml in 12 well plates (1 ml/well). 48 hours later, cells were counted using a hemocytometer. The protocol for electroporation is described in “qRT-PCR and RNA sequence analysis” Methods section. ***, p < 0.001.


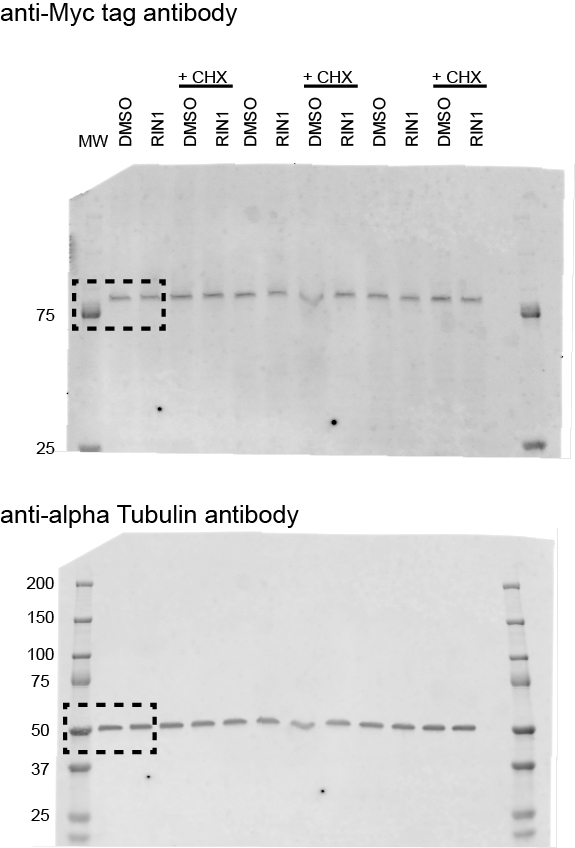
**Uncropped version of western blot in Fig. 1l. Dashed boxes indicate regions shown in the display figure.**

**Uncropped version of western blot in Fig. 2d. Dashed box indicates region shown in the display figure.** Note that the three proteins were detected independently on the same filter using infrared labelled secondary antibodies by the LiCor Odyssey instrument were superimposed to create the image shown (Merge). Each channel is shown separately below.

Merge

**
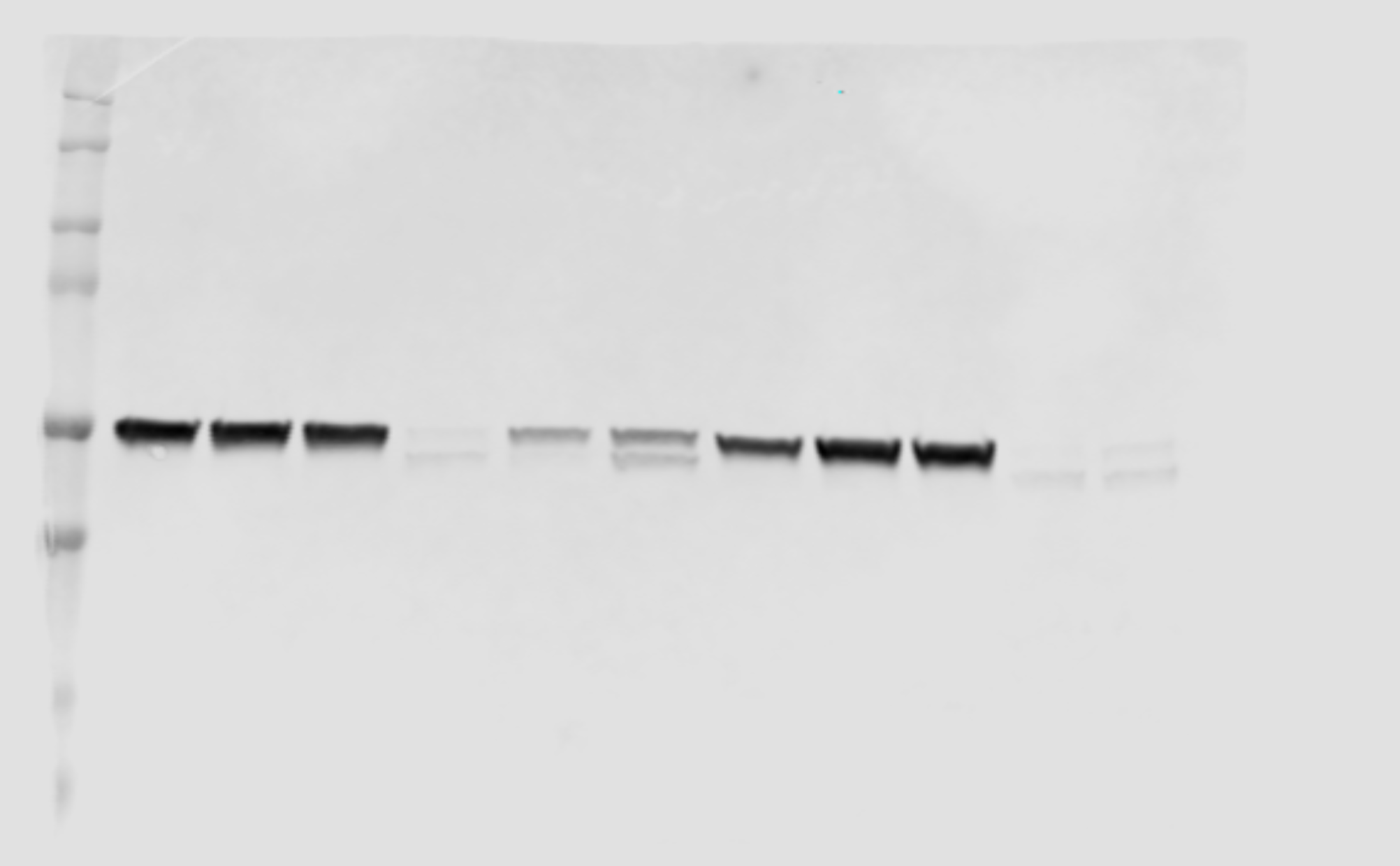

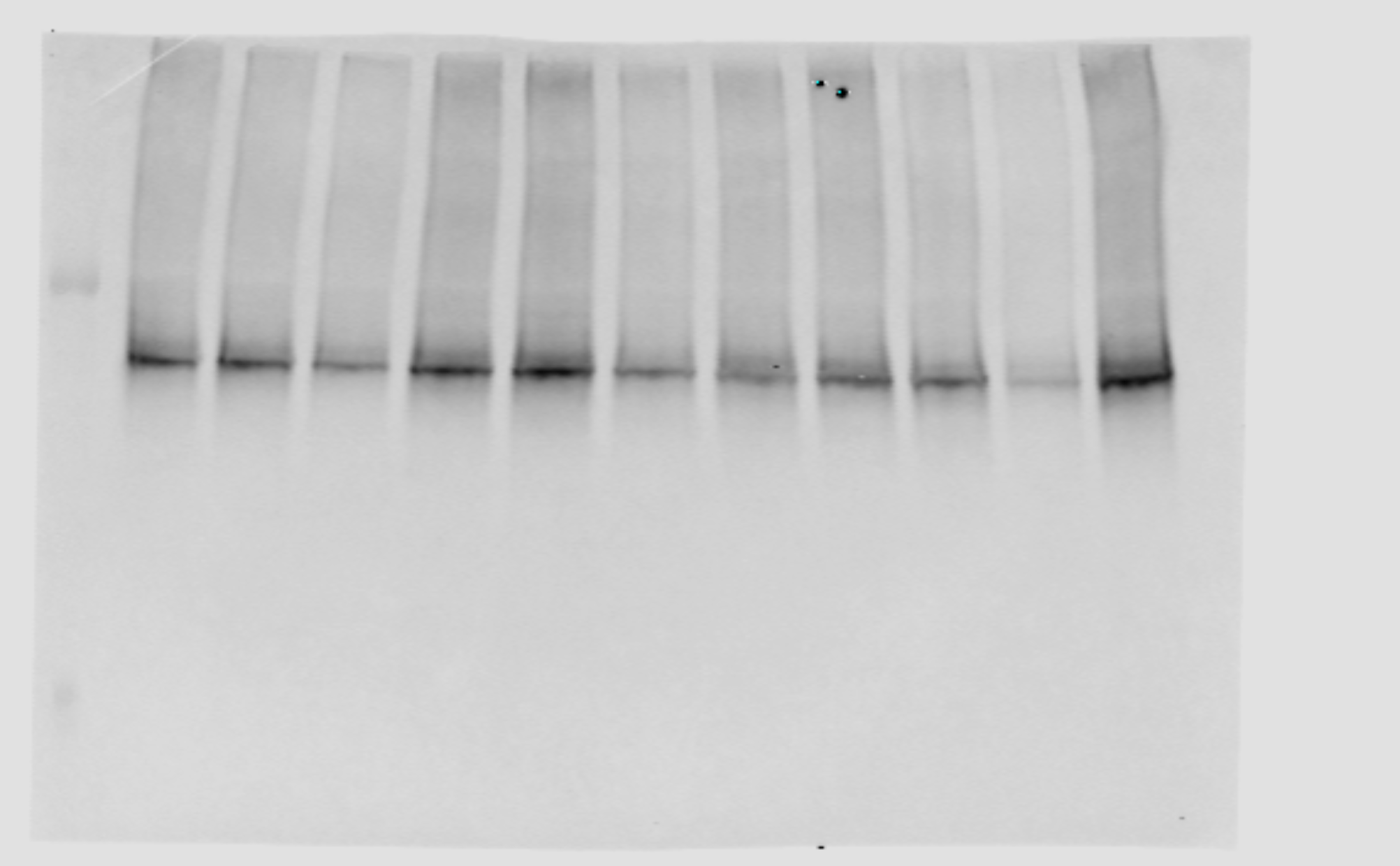
**RBPJ α-tubulin

**
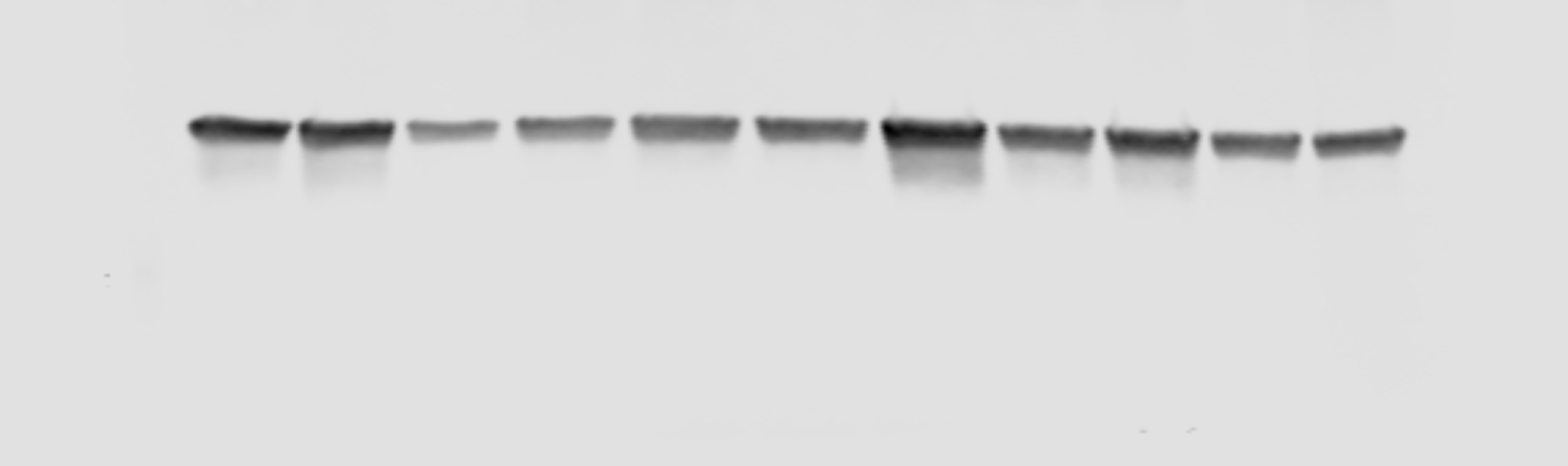
**GAPDH

**Uncropped version of western blot in Fig. S1. Dashed boxes indicate regions shown in the display figure.**
